# Editorial: Integrating health-related quality of life in neuro-oncology

**DOI:** 10.3389/fonc.2025.1573696

**Published:** 2025-03-18

**Authors:** Maria Teresa Pedro, Martina Messing-Jünger, Christine Jungk, Peter Hau, Carolin Weiss Lucas

**Affiliations:** ^1^ Peripheral Nerve Unit, Department of Neurosurgery, University Medicine of Ulm, Günzburg, Germany; ^2^ Pediatric Neurosurgery, Asklepios Children´s Hospital, St. Augustin, Germany; ^3^ Department of Neurosurgery, Heidelberg University Hospital, Heidelberg, Germany; ^4^ Department of Neurology and Wilhelm Sander NeuroOncology Unit, University Hospital Regensburg, Regensburg, Germany; ^5^ Centre of Neurosurgery, University Hospital of Cologne, Cologne, Germany

**Keywords:** HRQoL, mental health, distress, brain tumor, glioma, pediatric tumors, peripheral nerve tumor

This present Research Topic includes eleven articles contributed by 70 authors. Our aim was to give an up-to-date overview of health-related quality of life (HRQoL) in adult and pediatric patients spanning diverse neuro-oncological diseases. The HRQoL-related articles of the Research Topic provide a multifaceted overview of HRQoL-related topics, ranging from research on patients with brain metastases and gliomas to peripheral nerve tumors. Here, we summarize a selection of these articles.

The study “Psycho-oncological burden in patients with brain metastases undergoing neurological surgery” by Araceli et al. demonstrates the high prevalence of psycho-oncological distress among patients with surgically treated brain metastases (BM). The use of the Hornheider screening instrument (HSI) and Distress Thermometer (DT) allowed for the identification of a significant percentage of patients needing immediate intervention. The study further detected independent risk factors for high psycho-oncological burden, such as synchronous BM, female gender, and low KPS. This study emphasizes the need for routine psycho-oncological screening and interventions and the importance of addressing these psychological needs to improve overall BM patient care. The identification of specific risk factors allows for more targeted screenings and interventions.

In line with this, Staub-Bartelt et al., in their study “Influence of neuropathological diagnosis on psychooncological distress in neurooncological patients - a retrospective cross-sectional analysis,” address the psycho-oncological impact of glioblastoma (GB) as a life-changing disease. Although overall distress levels were similar between GB and grade 2 glioma patients, GB patients experienced significantly higher levels of depression. This highlights that even with similar overall distress, the ability to cope with specific mental health challenges depends on the type of tumor diagnosis. Using validated and robust scales (Hospital Anxiety and Depression Scale, Karnofsky Performance Score), the authors showed not only higher depression but also significantly greater physical impairment in GB patients. This study emphasizes the importance of early screening for depression and the need for targeted interventions tailored to the specific mental health needs of GB patients, in the context of comprehensive care beyond mere tumor treatment.

The study “Sexual life in adults treated for brain tumors: a retrospective study” by Leonetti et al. addresses a previously largely neglected aspect of HRQoL: While objectifiable sexual dysfunction is relatively uncommon, a substantial portion of patients experience a subjective decline in sexual well-being, often linked to relationship changes and treatment side effects. Based on its considerable impact on HRQoL, this study advocates more comprehensive HRQoL assessments and suggests the development of appropriate interventions to improve patients’ sexual well-being.

With their study on the “Preoperative subjective impairments in language and memory in brain tumor patients”, Rybka et al. provide important insights into the impact of cognitive difficulties on patients’ daily lives and emphasizes the need for assessments measures of cognitive function.

Duffau extend the field of attention towards creative functions, presenting an illustrative case of a drastic and largely inconscient change of creative behavior after resection of a right frontal low grade oligodendroglioma, titled “When art is faced with brain surgery: acute change in creative style in a painter after glioma resection”.

Especially the last-mentioned studies highlight the trend towards a progressively more comprehensive view on determining the functional limits of resection, most importantly in low grade glioma patients. This paradigm change might prove at least similarly meaningful in pediatric populations which suffer heavily from long-term treatment effects on quality of life, as Joh-Carnella et al. impressively illustrate with their case report titled “Pediatric low-grade gliomas: a fine balance between treatment options, timing of therapy, symptom management and quality of life”. The case unravels the complex challenge of managing low grade gliomas in children, highlighting the need for individualized treatment strategies and comprehensive, multidisciplinary care to balance disease control against the risk of long-term toxicity and, hence, symptom burden. Further research is needed to improve the prediction of treatment-related long-term effects and to optimize treatment strategies with regard to patient well-being in pediatric populations.

Sperl at al. systematic review shows that skull base tumor surgery temporarily reduces quality of life (QoL), which usually recovers. QoL is significantly affected by patient age, gender, tumor characteristics, surgical approach, resection extent, and pre-operative status. Radiotherapy and recurrent surgeries worsen QoL. Personalized care and early psychological intervention are crucial for optimal outcomes.

Savic et al. highlight the importance of individualized treatment for patients with peripheral nerve tumors (PNTs) due to their varied presentations. Grübel et al.’s large-scale, multicenter study (“Health-related quality of life in patients with peripheral nerve tumors: results from the German multicentric Peripheral Nerve Tumor Registry”) demonstrates that early surgical intervention at specialized centers significantly improves health-related quality of life (HRQoL). This improvement is seen across various subdomains, including pain relief, as measured by the EQ-5D-5L and EQ-VAS validated instruments. Therefore, early surgical treatment of PNTs is crucial for pain control and optimizing HRQoL.

Focusing more specifically on neurofibromatosis 1 (NF1), the study “Quality of life of patients with neurofibromatosis 1-Physical disability does not necessarily result in poor mental health” by Bäzner et al. suggests that symptomatic management should be considered even for severely affected patients to enhance their HRQoL.

In conclusion, the studies compiled in this Research Topic converge on several key topics: the importance of HRQoL assessments and interventions in neuro-oncology practice and science; the need for multidisciplinary approaches; the value of early interventions; and the role of specialized centers in improving outcomes. While each study focuses on a specific aspect, the overarching message is the crucial need to consider the overall patient integrity, addressing physical, psychological and social well-being alongside tumor control.

